# Beyond the embolus: “do not miss” diffusion abnormalities of ischaemic and non-ischaemic neurological disease

**DOI:** 10.1007/s13244-017-0574-1

**Published:** 2017-10-06

**Authors:** Vivek Yedavalli, Eric M. Nyberg, Daniel S. Chow, Ashesh A. Thaker

**Affiliations:** 1 Department of Diagnostic Radiology, Advocate Illinois Masonic Medical Center, Chicago, IL 60657 USA; 2 Department of Radiology, University of Colorado School of Medicine, Aurora, CO 80045 USA; 3 Department of Radiological Sciences, UC Irvine Health School of Medicine, Orange, CA 92868 USA

**Keywords:** Diffusion magnetic resonance imaging, Stroke, Neuroimaging, Hypoxia-ischaemia, Brain, Neurotoxicity syndromes

## Abstract

**Abstract:**

Given the rapid evolution and technological advances in the diagnosis and treatment of acute ischaemic stroke (AIS), including the proliferation of comprehensive stroke centres and increasing emphasis on interventional stroke therapies, the need for prompt recognition of stroke due to acute large vessel occlusion has received significant attention in the recent literature. Diffusion-weighted imaging (DWI) is the gold standard for the diagnosis of acute ischaemic stroke, as images appear positive within minutes of ischaemic injury, and a high signal-to-noise ratio enables even punctate infarcts to be readily detected. DWI lesions resulting from a single arterial embolic occlusion or steno-occlusive lesion classically lateralise and conform to a specific arterial territory. When there is a central embolic source (e.g. left atrial thrombus), embolic infarcts are often found in multiple vascular territories. However, ischaemic disease arising from aetiologies other than arterial occlusion will often not conform to an arterial territory. Furthermore, there are several important entities unrelated to ischaemic disease that can present with abnormal DWI and which should not be confused with infarct. This pictorial review explores the scope and typical DWI findings of select neurologic conditions beyond acute arterial occlusion, which should not be missed or misinterpreted.

***Teaching points*:**

• *DWI abnormalities due to acute arterial occlusion must be promptly identified.*

• *DWI abnormalities not due to arterial occlusion will often not conform to an arterial territory.*

• *Several important non-ischaemic entities can present on DWI and should not be confused with infarct.*

## Introduction

Although diffusion-weighted imaging (DWI) is most commonly relied upon for the detection of acute ischaemic infarct due to arterial large vessel occlusion (LVO), a wide variety of pathologies may result in abnormal DWI signal. The DWI signal increases when the random (Brownian) diffusivity of water molecules is reduced or restricted. The magnitude of diffusion can be quantified by calculating the apparent diffusion coefficient (ADC), where lower values represent areas of reduced diffusion [1]. In the setting of acute ischaemic infarct, energy metabolites are depleted, resulting in failure of membrane ion pumps, with extracellular water following the influx of sodium into the intracellular space. This leads to cytotoxic oedema, a pathological process defined by intracellular expansion due to osmotic shifts. With water molecules effectively trapped in the intracellular space, the ADC decreases, resulting in a hyperintense DWI signal. Any cellular process involving derangement of the physiologic exchange of water across cell membranes, regardless of whether the process leads to neuronal demise and apoptosis, can result in cytotoxic oedema and abnormal signal on DWI. For more detailed descriptions of cytotoxic oedema and the physical principles of DWI, we direct readers to comprehensive reviews by Simard et al. and Hagmann et al., respectively [2, 3].

When DWI abnormalities do not conform to a vascular territory or the clinical presentation is uncertain, a variety of aetiologies should be considered. The purpose of this pictorial review is to demonstrate typical DWI findings of common neurologic entities beyond acute arterial occlusion, which should not be missed or misinterpreted. Note that the entities presented here are not all-encompassing, but chosen to highlight frequently encountered conditions in which there may be clinical uncertainty, and management differs significantly from LVO.

## Hypoxic ischaemic encephalopathy

Global anoxic injury and hypoxic ischaemic encephalopathy (HIE) result from decreased oxygen delivery, with or without adequate perfusion, respectively. This can take a variety of forms based on the stage of brain development and the mechanism and duration of injury. Cardiac arrest and drug overdose can result in global ischaemia due to hypoperfusion, whereas drowning, status epilepticus, and respiratory failure can result in anoxia while the brain remains perfused [4]. While both anoxia and ischaemia result in neuronal death and cytotoxic oedema, this may occur more rapidly in hypoxia due to impaired clearance of toxic metabolites.

**Fig. 1:**
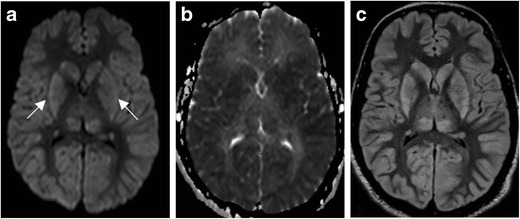
Hypoxic ischaemic encephalopathy. Sixty-eight-year-old man with prolonged cardiac arrest presenting with fixed and dilated pupils. DWI (**a**) demonstrates symmetric increased signal within the deep grey matter (*arrows*) and cerebral cortex. ADC (**b**) demonstrates corresponding low signal, consistent with reduced diffusivity. FLAIR (**c**) demonstrates corresponding symmetric increased signal throughout the deep grey matter and cerebral cortex

HIE typically presents with profound neurologic changes, including depressed mental status, seizure, and coma, with or without focal neurologic deficits. MRI findings vary, as any neural structure can be affected; however, certain findings are characteristic (Fig. 1). The salient point in these cases is that structures with the greatest metabolic demand at baseline will be most sensitive to injury when metabolites are scarce or absent. Grey matter is more metabolically demanding than white matter and is preferentially affected, distinct from LVO. In the acute phase (within hours of the insult), symmetric gyriform cortical reduced diffusion, often pronounced in the occipital lobes with additional involvement of the cerebellum and deep grey structures, is commonly identified [4]. As with other causes of cytotoxic oedema, DWI is positive first, followed by fluid-attenuated inversion recovery (FLAIR) imaging. T1 and FLAIR imaging may demonstrate symmetric cortical laminar necrosis in the subacute phase. This results in overall accentuation of grey-white matter differentiation. White matter reduced diffusion may not appear until the subacute phase. Outcomes tend to be poor, but may be better if cortical structures are spared [5].

## Hypoglycemic encephalopathy

**Fig. 2:**
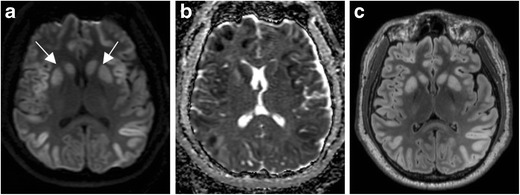
Hypoglycemia. Thirty-four-year-old man with altered mental status. DWI (**a**) demonstrates symmetric increased signal within the basal ganglia (*arrows*) and cerebral cortex, notably the insular and parietal cortex. ADC (**b**) demonstrates corresponding low signal, consistent with reduced diffusivity. FLAIR (**c**) illustrates corresponding symmetric increased signal within the basal ganglia and cerebral cortex. All sequences demonstrate notable sparing of the thalami

Hypoglycemia is defined as a serum glucose level of less than 50 mg/dL, and can present with a range of non-specific symptoms including weakness, altered mental status, seizures, and coma [6, 7]. As the brain is an obligate glucose metaboliser, injury can occur when the supply of blood glucose is not adequate to meet metabolic demand. This can result in partial failure of Na^+^/K^+^-ATPase pumps, resulting in cytotoxic oedema, and this imbalance can be exacerbated by the release of aspartate, an excitatory neurotransmitter. This can lead to neuronal necrosis, often affecting the cerebral cortex and basal ganglia, and often sparing the thalami, brainstem, and cerebellum (Fig. 2). However, imaging presentation varies, and white matter abnormalities in the internal capsules and corona radiata have also been reported [6, 8]. Imaging findings may appear similar to Creutzfeldt-Jakob disease and HIE; however, the former entity has a different clinical presentation and the latter often demonstrates more prominent occipital involvement [7, 9]. Diagnosis of hypoglycemic encephalopathy is straightforward once a glucose level has been obtained. Imaging is often helpful for prognosis, with one report suggesting that involvement of two or more lobes is associated with only partial recovery or death, whereas involvement of one lobe is associated with complete recovery [7].

## Carbon monoxide poisoning

Carbon monoxide poisoning is an anoxic encephalopathy caused by inhalation of carbon monoxide (CO). CO is known to have an affinity for haemoglobin (Hb) that is nearly 200 times that of oxygen, leading to an anoxic or hypoxic state. Its greater affinity for Hb results in decreased overall binding of oxygen and prevents bound oxygen from being released. CO also causes the release of free radicals and catabolic enzymes that can precipitate apoptosis. Presentation varies by dose, but generally includes a nonspecific prodrome of nausea, vomiting, and confusion [10].

**Fig. 3:**
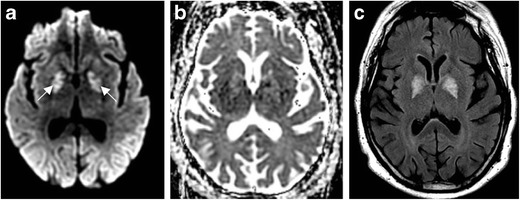
Carbon monoxide poisoning. Sixty-one-year-old man with history of substance abuse found unconscious. DWI (**a**) symmetric increased signal within the globus pallidus. ADC (**b**) demonstrates subtle corresponding increased signal, suggesting mildly increased diffusivity. FLAIR (**c**) illustrates corresponding symmetric increased signal within the globus pallidus

Imaging manifestations of CO poisoning include necrosis and reduced diffusion within the globus pallidus, with corresponding abnormalities on FLAIR becoming conspicuous in the subacute phase (Fig. 3). Decreased ADC may persist for weeks post exposure into the delayed phase. Putamina and caudate nuclei may be affected. Heterogeneous signals on T1- and T2-weighted images arising from blood products may be present in subacute and chronic phases, and enhancement varies. The involvement of white matter of the centrum semiovale and periventricular regions, which manifests as a high signal on FLAIR, is associated with worsened cognitive function. MR spectroscopy may demonstrate increased choline and decreased NAA, indicating active metabolism and demyelination, respectively [10]. Cortical involvement is uncommon, but may rarely occur with involvement of the temporal lobes and insula.

## Venous infarct

Venous infarction carries a high risk of morbidity and mortality, and can occur when venous egress from the brain is compromised. This entity most commonly occurs in the setting of cortical vein and/or dural venous sinus thrombosis. High flow shunt lesions resulting in venous hypertension can also result in venous infarct. Intracranial haemorrhage occurs more frequently in the setting of venous infarction compared with arterial infarct, possibly because arterial input pressure continues unabated in the setting of a compromised blood brain barrier. Systemic anticoagulation is the mainstay of treatment [11, 12], even in the setting of haemorrhage, and endovascular therapy with thrombolysis and/or thrombectomy [13, 14] of the dural venous sinuses has emerged as a therapeutic option.

**Fig. 4:**
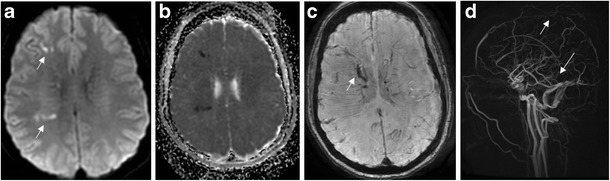
Venous infarct. Forty-three-year-old woman with headache and increased somnolence. DWI (**a**) and ADC (**b**) shows punctate foci of reduced diffusion in the right centrum semiovale (*arrows*). Susceptibility-weighted imaging (SWI) (**c**) demonstrates linear susceptibility along callososeptal veins, right greater than left (*arrow*). Magnetic resonance venography (MRV) (**d**) demonstrates loss of flow-related enhancement in the superior sagittal sinus and expected location of the internal cerebral veins, vein of Galen, and straight sinus (*arrows*)

DWI findings vary in the setting of venous compromise, but typically demonstrate areas of reduced diffusion within affected parenchyma and occasionally evidence of cortical venous thrombus (Fig. 4). Signal abnormality on DWI images in the setting of venous infarct may not be as intense as in arterial infarct [15, 16]. Reversible diffusion restriction has also been described. One possible explanation for this is that increased venous pressure results in compromised capillary tight junctions, with leakage of water into the extracellular space. Whereas in other settings, e.g. tumour, vasogenic oedema is typically unrestricted with increased ADC, in this setting extracellular water has no ready drainage pathway, and this gives rise to stasis and decreased diffusion [17]. This can be reversed as venous flow is restored. If, however, derangements in arterial inflow and accumulation of toxic metabolites result in cytotoxic oedema and cell death, this DWI signal would not be reversible.

## Creutzfeldt-Jakob disease

Creutzfeldt-Jakob disease (CJD) is a rare and devastating spongiform encephalopathy caused by cerebral deposition of aberrant infectious proteins known as prions. The sporadic form of CJD represents approximately 85% of all human prion diseases, which have an incidence of <1 per 100,000. CJD presents with a broad spectrum of clinical manifestations, often as a rapidly progressive dementia with other symptoms including visual disturbances, dysphasia, ataxia, and myoclonus. Patients often present with CJD in the sixth or seventh decade of life [18].

**Fig. 5:**
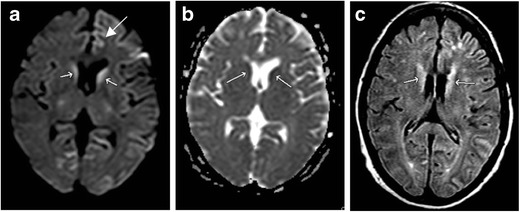
Creutzfeldt-Jakob disease. Forty-five-year-old man with limb weakness and tremors. DWI (**a**) demonstrates increased signal within the bilateral caudate heads (*small arrows*). There is also cortical increased signal within the left anterior cingulate gyrus (*large arrow*). ADC (**b**) demonstrates corresponding low signal within the caudate heads (*small arrows*). FLAIR (**c**) illustrates corresponding increased signal within the caudate heads (*small arrows*)

CT is often normal; however, MRI findings are well documented. Early DWI findings in sporadic CJD include high signal intensity in the cerebral cortex, caudate nucleus, putamen, and/or thalamus, with a distribution that does not correspond to the arterial circulation [19] and may be asymmetric (Fig. 5). The distribution of abnormal DWI follows a common pattern, involving mainly cortical areas near the midline, the insula, cingulum, and the superior frontal cortex [20]. One report suggests that sporadic CJD almost never involves only the limbic regions and rarely involves the precentral gyrus, patterns which may suggest non-prion aetiologies of rapidly progressive dementia [21]. FLAIR signal changes appear later, with signal changes on T2-weighted images often less conspicuous.

Variant CJD is more rare than sporadic CJD and is causally linked to bovine spongiform encephalopathy. The “pulvinar” sign, increased DWI signal within the posterior thalami, has been described as a highly accurate diagnostic sign, unique to the variant form of this condition [21, 22]. In suspected CJD, higher *b* values on DWI may make the subtle findings for this condition more conspicuous [23]. A uniform magnetic field and proper coil system are essential for increasing the conspicuity of subtle findings.

## Status epilepticus

Status epilepticus (SE) is the most debilitating form of epilepsy, resulting in a prolonged and contracted ictal state, with significant morbidity and mortality when left untreated. It is defined as a series of seizures without regaining consciousness for at least a 30-min period. The medial temporal lobe, specifically the hippocampus, is the most common location for epileptiform activity and is more vulnerable to excitotoxic injury [24]. The neocortex is also vulnerable to such injury [25]. Compared to arterial infarct, SE is the result of increased, not decreased, cerebral blood flow to vulnerable regions, due to overwhelming metabolic demand and consumption of oxygen. This eventually necessitates anaerobic respiration, with the accumulation of lactic acid, which in conjunction with vasodilation results in both vasogenic and cytotoxic oedema [25].

**Fig. 6:**
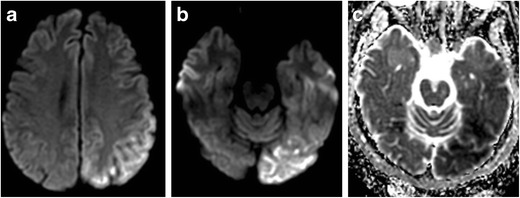
Status epilepticus. Forty-eight-year-old woman with seizures and altered mental status. DWI (**a**, **b**) demonstrates a predominantly increased signal with left parietal, occipital, and posterior temporal cortex. ADC (**c**) demonstrates a corresponding low signal consistent with reduced diffusion. Note that the area of signal abnormality spans both middle cerebral artery (MCA) and posterior cerebral artery (PCA) vascular territories

MRI is the mainstay of imaging for the detection of SE in both ictal and postictal states. An increased DWI signal is typically present within the cerebral cortex of the affected regions and may involve subcortical white matter (Fig. 6). The corresponding FLAIR signal abnormality is typically present, generally with no observable abnormal susceptibility or enhancement. In contrast to LVO, signal abnormalities do not usually conform to a single vascular territory, and an abnormal DWI/FLAIR signal, thought to represent transient vasogenic and cytotoxic oedema, should resolve upon short-interval follow-up imaging [25]. In cases of diagnostic uncertainty, arterial spin labelling (ASL) may be helpful in discriminating between status epilepticus and infarct, demonstrating elevated cerebral blood flow during ictal and early postictal states [26].

## Wernicke’s encephalopathy

Thiamine (vitamin B1) is critical to the formation of neurotransmitters, glucose metabolism, and myelin formation within the brain. The human body has thiamine stores that can last up to 6 weeks, but are quickly depleted without adequate nutrition. Wernicke’s encephalopathy (WE) is a metabolic derangement resulting from thiamine deficiency and is often associated with malnutrition in the setting of chronic alcoholism but can also be seen in malabsorption secondary to prior gastrointestinal surgery, underlying neoplasm, chemotherapy, or systemic infectious processes. WE is classically characterised by altered mental status, ocular dysfunction, and gait disturbances which are seen in up to 38% of patients. If thiamine is severely depleted, Korsakoff psychosis can also occur, which is characterised by severely altered mental status, confabulation, and personality changes. Approximately 80% of patients with alcohol abuse and WE eventually develop Korsakoff psychosis [27, 28].

**Fig. 7:**
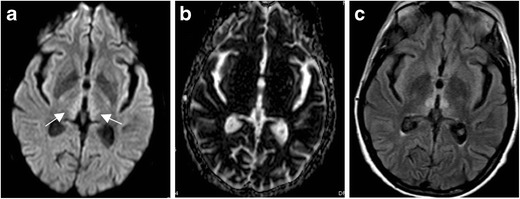
Wernicke’s encephalopathy. Sixty-two-year-old man with history of alcohol abuse and altered mental status. DWI (**a**) demonstrates increased signal within the medial thalami (*arrows*) and hypothalamus. ADC (**b**) demonstrates subtle corresponding increased signal, suggesting increased diffusivity. FLAIR (**c**) illustrates corresponding increased signal

Imaging findings in WE may be subtle. MR findings characteristically show a symmetric increased FLAIR signal within the medial thalamus (Fig. 7), as well as within periventricular areas of the third ventricle, mammillary bodies, and periaqueductal grey matter of the fourth ventricle. Contrast enhancement may occur in the affected regions, primarily within the thalami [27–30].

## Herpes simplex virus encephalitis

Herpes simplex virus (HSV) is a common sporadic viral encephalitis with significant morbidity and mortality if left untreated. The virus has two subtypes (HSV-1 and HSV-2), of which HSV-1 is responsible for 95% of encephalitides and occurs in adults and older children. HSV-2 predominates in neonates. HSV encephalitis (HSE) is more commonly due to reactivation of latent infection and is known to occur in immunocompromised states. It is thought that the virus spreads along the trigeminal nerve in a retrograde fashion into the leptomeninges of the medial cranial fossa, most often involving the temporal lobe [24, 31].

**Fig. 8:**
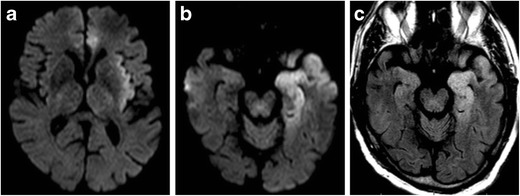
Herpes simplex virus encephalopathy. Seventy-one-year-old man with fever and encephalopathy. DWI demonstrates cortical increased signal within the left anterior cingulate and left insula (**a**) and the left mesial temporal lobe (**b**). FLAIR demonstrates corresponding increased signal (**c**). Note sparing of basal ganglia and thalami in (**a**)

HSE tends to present with a nonspecific prodrome of fever, headache, and altered mental status. Initial CT studies are often normal, or may demonstrate subtle temporal lobe hypodensity. MRI typically demonstrates an asymmetric abnormal signal within the medial temporal lobes, insular cortex, and cingulate gyrus, with abnormal DWI signal often the earliest abnormality (Fig. 8). Importantly, basal ganglia and thalami are typically spared, differentiating HSE from infarct due to proximal middle cerebral artery (MCA) occlusion [31]. Bilateral temporal lobe involvement is common, but usually asymmetric [32]. Gyriform petechial haemorrhage may be present on gradient-echo images with areas of cortical T1 shortening. Enhancement may be present in later phases and may be patchy or gyral. HSE has a 70% mortality rate when not promptly treated with IV acyclovir. Even with treatment, almost 97% of patients have significant residual neurological compromise [24].

## Transient lesion of the splenium

Transient lesion of the splenium (TLS), also known as mild encephalopathy with a reversible isolated splenial lesion (MERS), is a nonspecific, self-limiting entity seen in patients in a variety of clinical settings. Aetiologies include seizures, addition or cessation of antiepileptic drugs, and infectious encephalitis [33]. Numerous mechanisms have been proposed as potential causes of increased signal on DWI, including cytotoxic oedema, interstitial oedema, and swelling of the myelin sheath [34]. However, clinical and imaging findings of TLS typically demonstrate complete resolution, indicating that unlike oedema caused by infarct, the underlying pathophysiology of TLS is reversible.

**Fig. 9:**
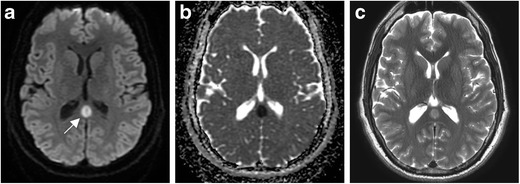
Transient lesion of the splenium. Twenty-seven-year-old man with epilepsy on anti-seizure medications, presenting with complex partial seizure. DWI (**a**) demonstrates a rounded focus of increased signal within the mid splenium. ADC (**b**) demonstrates corresponding low signal consistent with reduced diffusion. T2-weighted image (**c**) illustrates corresponding increased signal

TLS often manifests in one of two forms: a focal rounded lesion within the mid-splenium (Fig. 9) or a more irregular appearance with involvement of the majority of the splenium. The latter has been termed the “boomerang” sign, as it follows the contours of the splenium. These lesions generally appear low on T1 and demonstrate high signal on T2 and FLAIR without significant contrast enhancement. DWI is most sensitive with these lesions, exhibiting significantly reduced diffusion [35].

TLS may mimic an isolated infarct, which is a common lesion of the splenium. The condition can present with myoclonic seizures, tremors, general cognitive impairment, and bradykinesia. Time to resolution for TLS may vary from days to a year after clinical presentation. TLS is self-limiting, with treatment contingent upon its underlying cause, including resumption of antiepileptic drugs.

## Conclusion

Although DWI abnormalities are commonly associated with arterial large vessel occlusion, several other ischaemic and non-ischaemic processes should be considered when clinical or imaging findings are not consistent with arterial infarct. These include vascular and non-vascular hypoxic/anoxic conditions and metabolic and infectious encephalitides. Several of these entities have characteristic or pathognomonic imaging findings and should be readily recognised by practicing radiologists.
